# Intra-operative fluoroscopic radiation exposure in orthopaedic trauma theatre

**DOI:** 10.1007/s00590-017-2020-y

**Published:** 2017-08-10

**Authors:** Mustafa S. Rashid, Sheweidin Aziz, Syed Haydar, Simon S. Fleming, Amit Datta

**Affiliations:** 1 University of Oxford, Oxford, UK; 2 Broomfield Hospital, Chelmsford, UK; 3 Queens Hospital, Romford, UK

**Keywords:** X-ray, Fluoroscopy, Radiation, Exposure, Trauma operating room

## Abstract

**Introduction:**

Radiation exposure from intra-operative fluoroscopy in orthopaedic trauma surgery is a common occupational hazard. References for fluoroscopy use in the operating room for commonly performed operations have not been reported adequately. This study aimed to report appropriate intra-operative fluoroscopy use in orthopaedic trauma and compare the effect of surgery type and surgeon grade on radiation exposure.

**Methods:**

Data on 849 cases over an 18-month period were analysed retrospectively. Median and 75th centile values for dose area product (DAP), screening time (ST), and number of fluoroscopy images were calculated for procedures where *n* > 9 (*n* = 808).

**Results:**

Median DAP for dynamic hip screws for extracapsular femoral neck fractures was 668 mGy/cm^2^ (ST 36 s), 1040 mGy/cm^2^ (ST 49 s) for short proximal femoral nail, 1720 mGy/cm^2^ (ST 2 m 36 s) for long femoral nail for diaphyseal fractures, 25 mGy/cm^2^ (ST 25 s) for manipulation and Kirschner wire fixation in distal radius fractures, and 27 mGy/cm^2^ (ST 23 s) for volar locking plate fixation in distal radius fractures. These represented the five commonest procedures performed in the trauma operating room in our hospital. Experienced surgeons utilized less radiation in the operating room than junior surgeons (DAP 90.55 vs. 366.5 mGy/cm^2^, *p* = 0.001) and took fewer fluoroscopic images (49 vs. 66, *p* = 0.008) overall.

**Conclusions:**

This study reports reference values for common trauma operations. These can be utilized by surgeons in the operating room to raise awareness and perform clinical audits of appropriate fluoroscopy use for orthopaedic trauma, using this study as guidance for standards. We demonstrated a significant reduction in fluoroscopy usage with increasing surgeon experience.

## Introduction

Since the introduction of mobile fluoroscopy in trauma operating rooms in the early 1950s, their use has continued to be popular in modern trauma and orthopaedic surgery. The convenience of being able to use radiographs in real time without any delay during simple and complex fracture fixation comes at the cost of radiation exposure to the patient, surgeon, and allied health professionals assisting in the operative operating room.

In the UK, it is common practice for a mobile fluoroscopy (C-arm type) to be used for many fractures requiring operative management. These devices require a trained radiographer to work them, under the guidance of the operating surgeon. Everyone inside the operating room is required to wear protective lead apparel, and a thyroid shield is recommended. In cases involving young children, whereby the need for fluoroscopy is mandated, a lead protector is often used to cover the reproductive organs to limit radiation exposure. In the UK, all radiographers are required to wear radiation dosimeters that are checked at regular intervals to avoid excessive radiation exposure over time [1].

Currently, there are no set standards for patient radiation exposure in trauma operations requiring the use of a mobile fluoroscopy. Several studies have commented on radiation exposure for orthopaedic trauma procedures requiring fluoroscopy [2–4]. These are often limited by low numbers [4], incomplete data [2], or only focus on a subset of patients [3] and therefore are not applicable to most orthopaedic trauma cases treated in an average hospital. Radiation exposure remains a significant occupational hazard to the orthopaedic surgeon throughout their career [5]. It is good clinical practice to audit the radiation exposure to patients as well as medical staff including radiographers and orthopaedic surgeons [1]. Additionally, surgeons that are aware of the cumulative radiation dose in real time in the operating room adjust their behaviour to minimize this exposure [6]. However, surgeons are not informed as to what constitutes appropriate fluoroscopy use in terms of radiation dose, screening time, or number of images taken, for common orthopaedic trauma cases.

The aim of this study is to evaluate the patient radiation exposure in trauma operating rooms over an 18-month period in our hospital. Furthermore, we aim to determine reference values for common trauma procedures requiring fluoroscopy use. Analysis evaluating differences in radiation exposure between two operation types for certain fractures will be performed. Finally, we aim to analyse radiation exposure for all trauma cases by surgeon grade to determine any significant differences.

## Methods

Between 1 May 2013 and 1 October 2014, all trauma operations requiring mobile fluoroscopy were included for data collection. Using a standardized proforma, we utilized electronic operation note records (Bluespier, Droitwich, England) to determine the procedure type and surgeon grade. Electronic radiograph visualization software (AGFA Impax, Mortsel, Belgium) was used to determine cumulative dose per area (also known as dose area product, DAP), screening time (mm:ss), and number of X-rays taken intra-operatively from the dose report for each case. Data collection was performed retrospectively (by SA and SH). Statistical analysis was performed in SPSS version 24, IBM (by MR and SF).

Statistical analysis using the Mann–Whitney *U* test was applied to test for significant differences in DAP, screening time, and number of fluoroscopy images taken for all procedures where *n* > 9. The Mann–Whitney *U* test was used after a histogram plot demonstrated nonparametric data, and *p* < 0.05 was determined to be statistical significant. To determine reference values for fluoroscopy usage in the five commonest procedures, the 75th centile was calculated. The Mann–Whitney *U* test was applied to test for significant differences within DAP, screening time, and number of fluoroscopy images taken for distal radius fractures treated with open reduction internal fixation (ORIF using a volar locking plate) compared to those treated by manipulation under anaesthesia + Kirschner wire fixation (MUA + K-wires). Similar analysis was undertaken for intertrochanteric hip fractures treated with a dynamic hip screw (DHS) compared to short proximal femoral nail (PFN). Finally, utilizing the Mann–Whitney *U* test, differences in radiation exposure (DAP, screening time, and number of fluoroscopy images) were determined between consultant surgeons, specialty doctors, and orthopaedic specialist trainee registrars for all trauma cases.

Data on 849 cases were collected and available for analysis. Due to small numbers in less commonly performed operations, we excluded those where the frequency was *n* < 10 from statistical analysis, leaving 808 cases. The five commonest operations performed during the study period were dynamic hip screw for intertrochanteric proximal femoral fracture (190), MUA + K-wires of displaced distal radius fracture (139), short (240 mm) proximal femoral nail (PFN) for intertrochanteric proximal femoral fracture (75), ORIF with volar locking plate for displaced distal radius fracture (50), and long intra-medullary femoral nail for femoral fracture (39).

## Results

The 808 cases were included for statistical analysis (where *n* > 9) by operation type presented in Table 1. This case mix is felt to be representative of the fragility fracture population commonly encountered in a District General Hospital (DGH) in the UK. The median DAP, median screening time, and median number of fluoroscopy images taken for the cases were used to determine radiation exposure. The 75th centile reference values for DAP, screening time, and number of fluoroscopy images taken, for the five commonest operations performed are presented in Table 3. It was noted that intra-medullary (IM) femoral nail for subtrochanteric/diaphyseal femoral fractures carried the largest radiation dose with a median DAP of 1720 mGy/cm^2^. This requires further investigation to determine the cause for this significant radiation exposure, which is estimated to be 11 times a standard PA chest radiograph dose in our hospital.

**Table 1:** Frequency and type of trauma operations requiring fluoroscopy use including median dose area product, screening time, and number of fluoroscopy images taken for trauma cases where *n* > 9

	*n* =	Median DAP (mGy/cm^2^)	Median screening time (mm:ss)	Median no. of fluoroscopy images
Neck of femur (DHS)	190	667.5	00:36	65
Distal radius (MUA + K-wires)	139	25.2	00:25	45
PFN (short)	75	1040	00:49	109
Distal radius (ORIF)	50	27.35	00:23	45
IM femur nail (long)	39	1720	02:36	243
Neck of femur (cannulated screws)	35	881	00:56	113
Bimalleolar (ORIF)	33	64.5	00:22	42
Neck of femur (MUA-THR)	29	186	00:08	12
Forearm (MUA)	24	11.05	00:09	17
Fibula (ORIF)	22	37.35	00:15	29
Tibia (ORIF)	19	185	00:43	84
Distal radius (MUA)	18	11.25	00:10	16
Ankle (MUA)	16	47.3	00:10	21
Tibia (IM nail)	14	359.5	01:44	202
Femur (ORIF)	14	613	01:14	133
Elbow (MUA + pinning supracondylar)	13	87	00:55	107
Forearm (ORIF)	12	24.35	00:18	34
Elbow (MUA)	12	33.85	00:11	17
Elbow (ORIF)	12	81.25	00:18	37
Medial malleolus (ORIF)	11	57	00:15	29
Knee (ORIF-tibial plateau)	11	225	00:26	52
Tibia/fibula (MUA)	10	37.85	00:16	33
Humerus (ORIF)	10	139.5	00:23	42
Total	808			

When comparing two operation types for displaced distal radius fracture, ORIF and MUA + K-wires did not demonstrate significantly different median radiation exposure for DAP (27.4 vs. 25.2 mGy/cm^2^, *p* = 0.599), screening time (00:23 vs. 00:25 s, *p* = 0.362), and number of fluoroscopy images taken (45 vs. 45, *p* = 0.215). However, for intertrochanteric proximal femoral fractures, the two commonest operation types (DHS and PFN) demonstrated statistically significant differences in median DAP (668 vs. 1040 mGy/cm^2^, *p* < 0.001), median screening time (00:36 vs. 00:48 s, *p* < 0.001), and median number of fluoroscopy images taken (65 vs. 110, *p* < 0.001) (Table 2).

**Table 2:** Reference radiation exposure values (median and 75th centile) for five commonest trauma operations using dose area product, screening time, and number of II images taken

	*n* =	Median DAP (mGy/cm^2^)	75th centile DAP (mGy/cm^2^)	Median screening time (mm:ss)	Median no. of fluoroscopy images	75th centile no. of fluoroscopy images
Neck of femur (DHS)	190	667.5	20.2	00:36	65	88.5
Distal radius (MUA + K-wires)	139	25.2	0.43	00:25	45	69
PFN (short)	75	1040	24.45	00:49	109	143
Distal radius (ORIF)	50	27.35	0.47	00:23	45	58
IM femur nail (long)	39	1720	45.3	02:36	243	290.5

When considering the grade of surgeon, there were significant differences in radiation exposure between consultant, staff grade and associate specialist doctors (SAS), and specialist training registrars (StR). All cases were included and median patient radiation exposure was calculated. Consultants were first operating surgeons in 15.6% (*n* = 133) of trauma cases requiring fluoroscopy, SAS doctors in 50.6% (*n* = 429) of cases, and StR doctors in 33.8% (*n* = 287) of cases. The median DAP between consultant surgeons (90.6 mGy/cm^2^) and SAS doctors (175.5 mGy/cm^2^) was significant (*p* = 0.034). This was the case for median number of fluoroscopy images taken (49 vs. 66, *p* = 0.042); however, statistical significance was not demonstrated between consultants and SAS doctors for median screening time (00:26 vs. 00:32 s, *p* = 0.059). A comparison between consultant surgeons and specialist training registrars (StR) revealed a similar trend demonstrating significant difference in median patient radiation exposure. Median DAP was lower when consultants were first operating surgeon compared to StR doctors (90.6 vs. 366.5 mGy/cm^2^, *p* = 0.001). Median screening time (00:26 vs. 00:36 s, *p* = 0.011) and median number of fluoroscopy images taken (49 vs. 66, *p* = 0.008) also demonstrated statistically significantly less patient radiation exposure with consultant surgeons compared to specialist trainees.

For ease of clinical application, we recommend the 75th centile of number of fluoroscopic images as a marker for appropriate fluoroscopy usage in the operating room. Specifically, for DHS procedures there are 89 fluoroscopic images and 69 for MUA + K-wires in distal radius fixation. Other reference values for the five commonest procedures are given in Table 2.

## Discussion

Occupational radiation exposure to orthopaedic surgeons has been shown to increase the likelihood to develop a cancer during their lifetime compared to other healthcare workers by fivefold [7]. Methods employed to reduce the risk from ionizing radiation include: minimizing radiation use, personal protective apparel, routine radiation dose monitoring, and intra-operative positioning reducing scatter radiation [8, 9] (Table 3).

**Table 3:** Evidence-based recommendations for surgeons, radiographers, and institutions for minimizing ionization radiation exposure in conventional fluoroscopy in trauma operating room

Personnel responsible	Recommendation	Reference
Surgeons	Reduce number of repeat fluoroscopic images required by consideration and clear communication No continuous fluoroscopy unless absolutely necessary Ensure adequate patient positioning to facilitate use of intra-operative fluoroscopy Raise awareness of radiation exposure during intra-operative fluoroscopy to junior surgeons	[7–10]
Radiographers	Limitation of field size to area of interest (collimation) Reduce exposure timer to minimum Utilize highest practicable wattage and lowest amplitude Ensure no staff in direct line with path of primary beam Ensure doors to operating room closed Always wear dosimeter and regular monitor personal exposure	[1, 8, 9]
Institutions	Provide and maintain personal protective equipment including lead gowns, thyroid shields, and eye goggles Regular audit of radiation exposure of radiographers and high-risk individuals Limit staff in operating room using fluoroscopy to those only essential personnel Regular maintenance of fluoroscopic equipment	[1]

In our hospital, one radiographer is allocated to trauma operating room each day from a cohort of trauma-competent radiographers. This small group of radiographers is well known the orthopaedic department, and this aids in effective communication during operative cases, vital for effective fluoroscopy usage. Radiographers utilize the ALARA principle, which is strictly applied in operating rooms. This is an acronym for as low as reasonably achievable and involves adjustments based on body part, soft tissue window surface area, minimal screening time, and positioning of the detector [9]. Chaganti et al. demonstrated, via a questionnaire, that there was a variation of correct nomenclature for the movements requested of the fluoroscopy. They suggested that a uniform language between surgeons and radiographers may lead to less confusion and potentially improve operating room time utilization, and possibly radiation exposure [10].

Personal protective equipment (PPE) is essential when working with ionizing radiation. Lead gowns, thyroid shields, and eye goggles (usually with a lead equivalence of 0.5 mm [9]) are recommended for operating room staff at all times.

Radiographers are required to wear dosimeters under the lead apron to measure total body dose that is regularly checked to ensure they are not exposed to more than 0.5 mSv/month [1].

Finally, C-arm positioning and distance from the X-ray course is important for reducing exposure to staff. Fluoroscopy scatter radiation follows the inverse square law, which suggests that moving from 1 m to 2 m from the C-arm reduces radiation exposure by a factor of four [9]. The X-ray source produces the greatest radiation exposure in a linear direction and so standing directly behind the X-ray generator or detector/intensifier also reduces exposure [9]. Nordeen et al. demonstrated a statistically significant difference in radiation exposure (measured in screening time) when the surgeon had control of when a fluoroscopy image was taken rather than the radiographer [11].

The Ionising Radiation (Medical Exposure) Regulations 2000 require “the employer to set diagnostic reference levels” for “interventional procedures” in quantities that can be measured such as DAP or screening time [1]. Another measure, coined the “personal dose equivalent”, is the operational quantity, defined by the International Commission on Radiation Units and Measurements (ICRU) for individual monitoring below a specified point on the body at an appropriate depth. Given our study was retrospective, we utilized DAP, as this was routinely recorded for each operative case in keeping with the above regulations.

To our knowledge, there are few standardized reference values for common trauma procedures requiring fluoroscopy, though an attempt was made by Pillai and Jain [2]. They retrospectively reviewed 1000 cases of emergency orthopaedic trauma in a hospital in Lanarkshire, Scotland. A random mix of cases were included in the study; however, they grouped their operations, either by diagnosis (hip fractures) or body part, e.g. “Elbow”. The median DAP was reported, and of note, the median DAP for femoral nail was 68.5 cGy/cm^2^ with a 75th gentile of 98.15 cGy/cm^2^. Hip fractures were not subdivided into implant type; therefore, direct comparison cannot be made. They did not set diagnostic reference levels for examinations by operation type as this was not appropriately categorized [2]. Another attempt by Roux et al. [12] involved seven minimally invasive trauma procedures being performed 15 times successively. These included MUA + K-wiring of distal radius fractures, distal radius ORIF, percutaneous tibial and femoral nailing (without distal locking), and short femoral IM nail for intertrochanteric fracture (without distal locking). Mean DAP for long femoral IM nailing mean DAP was 1197 mGy/cm^2^ for subtrochanteric fractures and 1843 mGy/cm^2^ for diaphysial femoral fractures. Our study showed, despite distal locking, similar DAP for long IM femoral nail (1720 mGy/cm^2^). Median DAP for short proximal femoral nail for intertrochanteric fractures (1040 mGy/cm^2^) was slightly higher in our study (794 mGy/cm^2^). Distal radius MUA + K-wires required less median DAP in our study (25.2 mGy/cm^2^) compared to Roux et al. [12] (59.6 mGy/cm^2^), similarly for distal radius ORIF (27.35 vs. 38.4 mGy/cm^2^). This study demonstrates the variation which can occur within operative cases, which further highlights the need for local and national references for each procedure requiring fluoroscopy.

We found a lack of difference in radiation exposure for two distal radius fracture treatment modalities. Recently, a multicentre pragmatic randomized controlled trial (DRAFFT) demonstrated that displaced distal radius fracture that is reducible is just as effectively treated with MUA + K-wires or volar locking plate based on patient-reported outcome measures [13]. This study demonstrated equally low doses of radiation exposure in both treatment methods. There have been several studies attempting to demonstrate superiority of proximal femoral nailing over dynamic hip screw (DHS) fixation for intertrochanteric hip fractures [14]. These have focussed on a number of parameters including, bleeding, transfusion rates, complications, metalwork failures, reoperations, surgical time, and mortality. Our results demonstrate that a DHS produces significantly less radiation exposure compared to proximal femoral nailing (excluding reverse oblique fractures, which are all treated with intra-medullary fixation). This may be due to the relative lack of experience with DHS being done far more commonly than PFN. In the United Kingdom, the National Institute for Health and Care Excellence (NICE) recommends dynamic hip screw (DHS) as the preferred method of fixation for intertrochanteric proximal femoral fractures [15].

Our study adds some key findings that are of note for surgeons utilizing fluoroscopy for orthopaedic trauma surgery. Firstly, we present standard reference values for common orthopaedic trauma procedures requiring fluoroscopy. To our knowledge, there are only two studies that have attempted to report this. Patel et al. [3] reported screening time and DAP of 782 paediatric trauma cases utilizing fluoroscopy in the operating room. Salvia et al. studied the radiation exposure in 80 orthopaedic cases (16 different surgery types). This study was limited by low numbers of cases per surgery type (only distal radius volar lock plate fixation had more than nine cases). Additionally, only screening time was measured in this study [4]. Our study describes DAP and screening time for a larger number of orthopaedic trauma cases, and with a greater number of cases per surgery type, hence increasing the validity of our findings. We found that surgeon experience correlated with radiation exposure. This is in line with the study by Patel et al. [3]. In paediatric orthopaedic trauma cases, they found that consultant surgeons used 51% less screening time and 35% less radiation exposure (as measured by DAP), than junior surgeons. Baumgartner et al. [6] demonstrated that orthopaedic surgeons change behaviour and reduce radiation exposure when they are made aware of radiation dose in the operating room. By reporting standard reference values for radiation exposure in common orthopaedic trauma cases, we believe surgeons will become more aware and consequently adapt behaviour to minimize this hazard in the operating room.

There are limitations with our study, as it is challenging to determine reference values for complex and variable interventions such as trauma operations. This study reports the patient radiation exposure for a variety of trauma operations; however, we did not use dosimeters to measure whether the differences in patient radiation exposure ultimately correlated with surgeon and assistant radiation exposure. The experience of the radiographer in trauma operating room is another confounder that could not be determined in this study. Fracture complexity is another confounder, with more complex fractures potentially requiring greater radiation exposure. We did not classify fractures based on configuration, complexity, or degree of comminution for example. In our hospital, the more complex fracture operations are usually undertaken by consultant surgeons, who, as a cohort, had the lowest fluoroscopy usage. This could mean that despite treating potentially more difficult cases, this was offset by surgeon experience, which reduced radiation exposure in the operating room overall. There is some bias in our study design; a retrospective cohort study did not allow us to measure and control for certain confounders as previously mentioned; however, our data are collected prospectively and reference values are representative of actual patient radiation exposure in our hospital during the study period. Similarly, for certain operation types, there were small numbers precluding meaningful statistical analysis; hence, these (where *n* < 10) were excluded. Despite these limitations, we present findings consistent with previously published literature but with larger numbers, and more consistency in reporting the type of operation performed and including surgeon grade.

Our study reports a correlation between surgeon experience and radiation exposure in the operating room. This is confounded by several factors including surgical complexity, fracture configuration, and radiographer experience, amongst others. Additionally, surgical experience does not necessarily equate to surgical competence. Hence, further work to investigate whether fluoroscopy usage can be used and validated as an independent marker for surgical competence in trauma surgery should be done.

## Conclusions

This retrospective cohort study illustrates patient radiation exposure for common trauma operations requiring mobile fluoroscopy performed in a District General Hospital in the UK. Reference values have been calculated for the five commonest procedures encountered and can act as standards for future audit. Dynamic hip screw fixation required less radiation exposure compared to short proximal femoral nail for intertrochanteric proximal femoral fractures in our hospital. Consultant trauma and orthopaedic surgeons used less radiation and took fewer fluoroscopic images during trauma cases compared to junior surgeons.
